# First report on leucocytes cell population data as an applicable test for clinical stratification in people living with HIV

**DOI:** 10.1016/j.virusres.2026.199716

**Published:** 2026-03-20

**Authors:** Haidi Karam-Allah Ramadan, Aml A. Rayan, Zeinab R. Mohamed, Mohammed Ezz-Eldin, Ahmed A. Kotb, Mohamed Ahmed, Rasha Assad Assiri, Salwa Seif Eldin, Amal A. Elkhawaga

**Affiliations:** a Department of Tropical Medicine and Gastroenterology, Faculty of Medicine, Assiut University, Assiut 71515, Egypt; b Department of Clinical Pathology, Faculty of Medicine, Assiut University, Assiut 71515, Egypt; c Department of Microbiology and Immunology, Faculty of Pharmacy, Assiut University, Assiut 71515, Egypt; d College of Medicine, Dhofar University, Salalah 211, Sultanate of Oman; e Department of Basic Medical Sciences, College of Medicine, Princess Nourah Bint Abdurahman University, Riyadh 11671, Saudi Arabia; f Department of Medical Microbiology and Immunology, Faculty of Medicine, Assiut University, Assiut 71515, Egypt

**Keywords:** HIV, Cell population data, Leucocyte size, Sysmex, Hematology analyzer

## Abstract

**Background:**

Leucocytes cell population data (CPD) parameters have been applied in diagnosis and prognosis of viral infections. We aim to explore pattern of leucocytes CPD in people living with HIV (PLWH) and its role in clinical stratification.

**Methods:**

This study included treatment-naïve PLWH and healthy control. Clinical HIV stages were determined. Laboratory assessment was conducted using complete blood picture, CPD, CD4+ *T* cells count, and HIV RNA testing. Correlation between CPD with CD4+ and HIV RNA was evaluated.

**Results:**

A total of 125 were recruited; 70 cases and 55 control. Age and gender were not significantly different. WHO stage 3 was predominant at 34.29% and half of PLWH had advanced HIV. CPD parameters of neutrophils, lymphocytes and monocytes composition were significantly higher in PLWH. Parameters of neutrophils nucleic acid contents, lymphocytes dispersion of nucleic acid contents, and both neutrophils and lymphocytes size were significantly higher in PLWH. CD4+ *T* cells count positively correlated with WBCs, lymphocytes and monocytes count and size, but negatively correlated with lymphocytes composition, nucleic acid contents, and size, and both monocytes and neutrophils composition and nucleic acid contents. Viral load negatively correlated with neutrophils size.

**Conclusion:**

CPD can be a cost-effective quantitative tool reflecting immune depletion in HIV. By decoding these parameters, clinicians can stratify PLWH risk of disease progression beyond CD4+ *T* cells count and viral load.

## Highlights

• CPD is an applicable test for clinical stratification in people living with HIV.
• Lower CD4+ count correlated with changes in size of lymphocytes and monocytes.
• CD4+ count negatively correlated with lymphocytes, monocytes and neutrophils composition and nucleic acid contents.
• CPD can be a cost-effective quantitative tool reflecting immune depletion in HIV.
• Decoding these parameters helps clinicians to stratify risk of HIV progression beyond CD4+ and viral load.

## Graphical abstract

All these sections are required for each article type.

**Figure:**
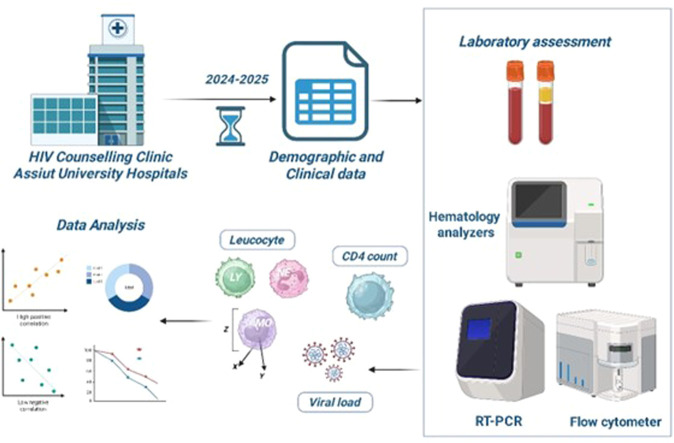

## 1 Introduction

Leucocytes cell population data (CPD) parameters, reported along with the complete blood picture (CBC), provide data on neutrophils (NE), lymphocytes (LY) and monocytes (MO) morphology and function. These parameters represent the size (NE-Z, LY-Z, MO-Z) nucleic acid content (NE-Y, LY-Y, MO-Y) and internal composition (NE-X, LY-X, MO-X) (Urrechaga et al., 2023). Leucocytes CPD are identified by three physical measurements; impedance method applied using direct current to measure cell volume, radiofrequency method using a high frequency current giving information on the nuclear size and density, laser beam light scatter to measuring cytoplasmic granularity (Grimaldi and Scopacasa, 1998; Picard et al., 1999).

Measurement of parameters such as neutrophils is based on the principles of fluorescence flow cytometry. Upon activation, cells change the composition of plasma membrane lipid particles and exhibit different cytoplasmic differentiation, resulting in higher fluorescence intensity (Rutkowska et al., 2024). The fluorescent agent combines with these structures and, under the influence of laser light, emits a higher intensity of light (Zeeshan-Haider et al., 2020). Neutrophilic cytoplasmic structure shows granulation with the nucleic acid components which increases as a result of cytokine synthesis (Urrechaga et al., 2023). Changes in leucocytes CPD were reported as a response to bacterial (Urrechaga et al., 2018) and viral infections (Zhu et al., 2013), or sepsis (Urrechaga, 2020).

Moreover, CPD parameters have been widely applied in early prediction and diagnosis of malaria (Buoro et al., 2018), leukemia (Haider et al., 2020; Boshnak and Zaiema, 2023), and acute pancreatitis (Wang et al., 2021), also used to stratify severity of sepsis (Mishra et al., 2025) and played a prognostic role in COVID-19 (Vasse et al., 2023).

In viral infection, the immune response is more dependent on activation of lymphocytes mainly T cells. Activated lymphocytes may undergo both morphologic changes, such as an increase in size, and changes in cytoplasmic composition as compared to their resting counterparts (Xu, 2015). These morphologic changes or in other terms CPD of reactive lymphocytes can be quantitatively detected using automated hematology analyzers technology (Silva et al., 2006).

Moreover, certain monocyte and lymphocyte CPD parameters have diagnostic and prognostic value for COVID-19 (Harte et al., 2023; Hossain et al., 2022). Morphological changes of lymphocytes and/or monocytes in hepatitis B virus (HBV) infection were observed and helped in identifying HBV infection (Zhu et al., 2011). Monocyte CPD has shown to be useful in differentiation between malaria and dengue infections on hematology analyzer using volume, conductivity, and scatter data for the monocytes (Sharma et al., 2014). Additionally, combining both elevated lymph index and the monocytes distribution width (MDW) with normal or mildly decreased total leucocytic count or lymphocyte count appear to be unique leucocyte profiles for viral infection (Zeng et al., 2020).

Human immunodeficiency virus (HIV) can enter cells by interaction between HIV envelop glycoprotein gp120 and the primary CD4+ cell membrane receptor (Chen, 2019). Therefore, CD4+ *T* cells count is considered the most important traditional biomarker for assessing HIV severity (Stebbing et al., 2005). HIV also triggers other innate immune responses involving neutrophils (Hensley-McBain and Klatt, 2018) and monocytes (Mensching and Hoelzemer, 2022).

A change in leucocytes CPD is expected with HIV, however, there is lack of studies on the pattern of CPD in HIV and its role in clinical stratification. Hence, the aim of this study was to evaluate the pattern of changes of leucocytes CPD in people living with HIV (PLWH) and its correlation with HIV clinical stages.

## 2 Materials and methods

This case control study was conducted on PLWH attending HIV Counselling Clinic, in Assiut University Hospital between 2024–2025. The patients included were treatment-naïve and were excluded if they were currently receiving antiretroviral therapy (ART). Healthy blood donors were selected as controls because they represent a well-screened reference population and were included only if they had a normal CBC, no suspicious analyzer flags, and normal peripheral blood smear findings. Demographic and clinical data were evaluated. Laboratory assessment was conducted using CBC, CD4+ *T* cells count, and HIV nucleic acid testing.

### 2.1 CBC and CPD parameters

Venous blood samples were collected in K₂-EDTA tubes and processed within 2 h on the Sysmex XN-1000 automated hematology analyzer (Sysmex Corporation, Kobe, Japan).

The XN-1000 utilizes fluorescence flow cytometry technology to evaluate leucocyte morphology. The analyzer was calibrated and maintained according to the manufacturer’s instructions (Sysmex XN-series Calibration Protocol), with daily internal quality control (3 levels of Sysmex XN Check) and External Quality Assurance program [College of American pathologist proficiency test (CAP PT)] to ensure the stability of both routine and structural (CPD) parameters.

Verification of the analyzer was performed according to the Clinical and Laboratory Standards Institute (CLSI) guideline H26-A2 for validation and verification of automated hematology analyzers to ensure the reliability of analyzer results (Clinical and Laboratory Standards Institute, 2019). Consistency with other XN-series models (e.g., XN-10/20) is supported by prior validation study by Seghezzi et al. (2018) showing high inter-instrument agreement, enabling comparable results across laboratories that follow standardized Sysmex protocols (Seghezzi et al., 2018).

Routine CBC parameters, including WBC count, hemoglobin, platelets, and differential counts, were retrieved along with leucocyte CPD indices. These CPD parameters are generated automatically during routine analysis without additional reagents or sample processing, allowing for an objective and reproducible assessment of morphological characteristics. Derived from the WBC differential fluorescence (WDF) channel, these indices represent cell size (Forward Scatter, FSC/Z-axis), internal complexity or granularity (Side Scatter, SSC/X-axis), and nucleic acid/protein content (Side Fluorescence, SFL/Y-axis). Specifically, the analyzer reported values for neutrophils (NE-SSC, NE-FSC, NE-SFL), lymphocytes (LY-X, LY-Y, LY-Z), and monocytes (MO-X, MO-Y, MO-Z). Additionally, the distribution width for each leucocyte subset: NE-WX, NE-WY, NE-WZ (neutrophils), LY-WX, LY-WY, LY-WZ (lymphocytes), and MO-WX, MO-WY, MO-WZ (monocytes), describe the dispersion or heterogeneity within each population. These width parameters are calculated based on the distribution range of the major cellular population, excluding outliers below 20% of the peak height in the distribution curve. The specific biological significance of each parameter is summarized in Supplementary Table 1.

### 2.2 CD4+ *T*-cell enumeration

CD4+ *T*-lymphocyte counts were determined for PLWH using flow cytometry. Peripheral blood samples were stained with monoclonal antibodies against CD4 (PE-Cyanine7, PC7) and CD45 (fluorescein isothiocyanate, FITC), obtained from Beckman Coulter (Brea, CA, USA), following the manufacturer’s protocol and acquired on CytoFLEX Flow Cytometer (Beckman Coulter, Brea, CA, USA). Absolute CD4+ *T* cells counts were calculated using a dual-platform method, which combined lymphocyte counts obtained from the CBC with CD4 percentages determined by flow cytometry.

### 2.3 Viral nucleic acid load testing

HIV RNA was quantified from EDTA plasma using the artus HI Virus-1 RG RT-PCR Kit (QIAGEN, Hilden, Germany) on the Rotor-Gene Q Real-Time PCR System (QIAGEN), following the manufacturer’s manual protocol, and results were expressed as IU/mL.

Statistical analysis*:* Data analysis was performed using SPSS version 23. Categorical variables were expressed as frequency and percentages. Continuous variables normally distributed were presented as mean ± SD and compared using *t*-test or presented as median and interquartile range (IQR) if not normally distributed and compared using Mann-Whitney test. Comparison between categorical variables was conducted using Chi test. Spearman’s correlation coefficient was calculated to assess the correlation between the variables of interest. P value was set at < 0.05 for statistical significance.

## 3 Results

### 3.1 Baseline characteristics and CBC data

A total of 125 participants were included; 70 cases and 55 control. Table 1 illustrates the baseline demographic and CBC data. Age and gender were not significantly different among both groups. WBCs, hemoglobin level, and lymphocyte counts were significantly lower in PLWH than healthy control. Platelets, neutrophils and monocytes counts were not significantly different between both groups. Median CD4+ *T* cells count was 193.95 cells/mm^3^ and viral load could be measured in 59 patients, and the median was 101,430 copy/ml. World Health Organization (WHO) stage 3 was predominant in 34.29%, while 28.57% had stage 1, 20% had stage 4 and 17.14% had stage 2. Advanced HIV defined as CD4+ *T* cells count <200 cells/mm³ or WHO stage 3 or 4 was detected in 40 (57.1%) (WHO, 2026).

**Table 1:** Baseline demographic characteristics and CBC data.

Items	PLWH (*n* = 70)	Control (*n* = 55)	*p* value
Age (years)	33.99 ± 8.48	32.38 ± 7.01	0.260
Sex: (n, %) Males Females	48 (68.57%) 22 (31.43%)	41 (74.55%) 14 (25.45%)	0.464
CD4+ *T* cells (cells/mm³) (Range)	193.95 (416.10) (2.1- 1208.9)	—	—
HIV viral load (IU/mL) (Range)	101,430 (108,214) (1554- 1000,000)	—	—
WBCs (x10^3^/ul)	5.48 ± 2.01	7.05 ± 1.68	0.000*
Hb (gm/dl)	11.68 ± 2.69	13.69 ± 1.80	0.000*
Platelets (× 10^3^/ul)	247.16 ± 90.11	272.42 ± 74.75	0.097
Neutrophils (× 10^3^/ul)	3.17 ± 1.72	3.66 ± 1.27	0.078
Lymphocytes (× 10^3^/ul)	1.58 (1.51)	2.42 (1.19)	0.000*
Monocytes (× 10^3^/ul)	0.59 (0.85)	0.58 (0.25)	0.788

Data are presented as (mean ± SD) or median (IQR). WBCs: white blood cells, Hb: hemoglobin, * Significant *p* value.

### 3.2 Description of CPD and data related to cell morphological characteristics

Parameters of neutrophils, lymphocytes and monocytes composition were significantly higher in PLWH compared to control. In addition, neutrophils nucleic acid compositions, lymphocytes WY, and both neutrophils and lymphocytes sizes were significantly higher in PLWH. LY-Z to MO-Z ratio was significantly higher in PLWH than control indicating more pronounced effect on lymphocytes size compared to monocytes size. On comparing CPD parameters between those who had CD4+ *T* cell count < 200 cells/mm^3^ and those with CD4+ count ≥200 cells/mm^3^, parameters of neutrophils, lymphocytes and monocytes composition, neutrophils nucleic acid contents, both neutrophils and lymphocytes sizes, and LY-Z to MO-Z ratio were significantly higher in those with CD4+ *T* cell count <200 cells/mm^3^ (Table 2).

**Table 2:** Comparison of cell population parameters regarding cell complexity, nucleic acid and cell size between PLWH and control, and according to CD4+ *T* cells count.

Cell population parameters	PLWH (*n* = 70)	Control (*n* = 55)	*P* value	CD4+ ≥200 cells/mm^3^ (*n* = 35)	CD4+ <200 cells/mm^3^ (*n* = 35)	*P* value
**X- axis** (**internal composition or cell complexity) (mean ± SD)**						
NE-SSC	152.95 ± 4.94	149.62 ± 4.99	0.000*	151.26 ± 4.55	154.63 ± 4.79	0.004*
LY-X	78.45 ± 3.20	77.13 ± 2.23	0.011*	76.69 ± 2.39	80.21 ± 2.95	0.000*
MO-X	118.78 ± 4.25	115.94 ± 1.91	0.000*	116.90 ± 3.65	120.67 ± 4	0.000*
NE-WX (Neutrophil fluorescence)	341.03 ± 47.44	334.27 ± 37.57	0.389	327.74 ± 44.25	354.31 ± 47.38	0.018*
LY-WX	561.08 ± 118.38	542.13 ± 111.02	0.363	563.77 ± 101.02	558.40 ± 132.68	0.851
MO-WX	286.69 ± 51.09	269.44 ± 21.84	0.021*	288.43 ± 61.69	284.94 ± 38.55	0.778
**Y- axis (cell nucleic acid) (mean ± SD)**						
NE-SFL	51.66 ± 6.14	46.41 ± 2.94	0.000*	48.78 ± 2.67	54.54 ± 7.23	0.000*
LY-Y	67.90 ± 7.17	68.28 ± 3.56	0.721	66.76 ± 2.24	69.04 ± 9.14	0.186
MO-Y	108.41 ± 13.25	106.08 ± 6.12	0.229	105.89 ± 11.75	110.94 ± 14.33	0.112
NE-WY (width of dispersion of neutrophil Fluorescence)	713.20 ± 134.82	688.44 ± 81.54	0.232	661.34 ± 44.91	765.06 ± 171.28	0.001*
LY-WY	1011.21 ± 176.89	935.20 ± 95.51	0.005*	1049.57 ± 87.62	972.86 ± 229.77	0.069
MO-WY	710.27 ± 207.62	737.40 ± 96.72	0.372	738.74 ± 112.62	681.80 ± 270.43	0.254
**Z- axis** (**cell size) (mean ± SD)**						
NE-FSC	85.58 ± 4.85	83.47 ± 4.68	0.016*	87.19 ± 4.51	83.97 ± 4.69	0.005*
LY-Z	57.94 ± 3.53	56.41 ± 1.47	0.003*	56.84 ± 189	59.01 ± 4.36	0.010*
MO-Z	63.15 ± 5.68	64.28 ± 2.75	0.179	63.64 ± 3.63	62.66 ± 7.2	0.473
NE-WZ (width of dispersion of neutrophil Size)	883.86 ± 106.69	900.73 ± 103.03	0.375	861.77 ± 101.74	905.94 ± 108.37	0.083
LY-WZ	808.77 ± 111.37	771.87 ± 66.26	0.032*	784.74 ± 50.93	832.11 ± 145.43	0.077
MO-WZ	911.78 ± 133.41	879.87 ± 78.92	0.119	909.24 ± 70.9	914.26 ± 175.23	0.877
LY-Z to NE-FSC ratio	0.68 ± 0.06	0.68 ± 0.04	0.894	0.65 ± 0.04	0.70 ± 0.06	0.000*
LY-Z to MO-Z ratio	0.93 ± 0.1	0.88 ± 0.04	0.003*	0.9 ± 0.05	0.96 ± 0.13	0.017*

NE: neutrophils, LY: lymphocytes, MO: monocytes, * Significant *p* value.

In Table 3, comparing CPD parameters between those who did not have opportunistic infections (*n* = 47) and those with opportunistic infections (*n* = 23) showed that parameters of neutrophils, lymphocytes and monocytes compositions, and neutrophils and monocytes nucleic acid contents were significantly higher in those with opportunistic infections, while no significant difference was detected regarding lymphocytes nucleic acid parameters or other cellular z- axis parameters (cell size).

**Table 3:** Comparison of parameters of CPD pattern between PLWH who did not have opportunistic infections and PLWH with co-infections.

CPD Parameters	HIV without opportunistic infection (*n* = 47)	HIV with opportunistic infections (*n* = 23)	P value
**X- axis** (**cell complexity) (mean ± SD)**			
NE-SSC (density or complexity of the cell and depicts the granularity)	152.1 ± 5.23	154.69 ± 3.8	0.038*
LY-X	77.79 ± 2.96	79.80 ± 3.32	0.013*
MO-X	117.96 ± 4.08	120.47 ± 4.18	0.019*
NE-WX (width of dispersion of neutrophil complexity)	340.79 ± 40.21	341.52 ± 60.64	0.952
LY-WX	564.68 ± 68.81	553.73 ± 184.3	0.719
MO-WX	288.77 ± 58.92	282.43 ± 30.01	0.630
**Y- axis (cell nucleic acid) (mean ± SD)**			
NE-SFL (Neutrophil fluorescence)	49.97 ± 4.5	55.11 ± 7.57	0.001*
LY-Y	66.85 ± 7.89	70.04 ± 4.88	0.080
MO-Y	105.91 ± 13.03	113.53 ± 12.45	0.023*
NE-WY (width of dispersion of neutrophil Fluorescence)	679.40 ± 77.82	782.26 ± 192.15	0.002*
LY-WY	1049.17 ± 127.98	933.65 ± 233.50	0.009*
MO-WY	744.96 ± 203.87	639.39 ± 201.18	0.045*
**Z- axis (cell size) (mean ± SD)**			
NE-FSC	**86±4.74**	84.72 ± 5.07	0.300
LY-Z	57.52 ± 2.88	58.77 ± 4.51	0.165
MO-Z	63.92 ± 5.55	61.57 ± 5.74	0.103
NE-WZ (width of dispersion of neutrophil Size)	875.25 ± 111.22	901.43 ± 96.73	0.339
LY-WZ	814.02 ± 113.57	798.26 ± 108.54	0.583
MO-WZ	907.59 ± 111.11	920.17 ± 172.19	0.715

### 3.3 Correlation between CD4+ *T* cell count, HIV viral load, and WHO clinical stage with CBC and CPD

As shown in Table 4, CD4+ *T* cells count positively correlated with WBCs, lymphocytes and monocytes count and size. Meanwhile, it was negatively correlated with lymphocytes composition, nucleic acid contents, and size, and both monocytes and neutrophils composition and nucleic acid contents. Viral load negatively correlated with neutrophils size only (*p* = 0.019). Significant negative correlation was observed between WHO clinical stages with lymphocytes and monocytes count. Meanwhile, significant positive correlation was reported between WHO clinical stages with neutrophils and monocytes composition and nucleic acid contents, and all parameters of lymphocytes

**Table 4:** Correlation between CD4+ *T* cell count, HIV viral load, and WHO clinical stages with CBC and CPD parameters.

Parameters		CD4+ *T* cell count	HIV Viral load	WHO stage
**WBCs**	R	0.481	0.034	-0191
	*p* value	0.000*	0.798	0.113
**Neutrophils**	R	0.039	0.044	0.115
	*p* value	0.719	0.740	0.342
**Lymphocytes**	R	0.853	-0.045	-0.557
	*p* value	0.000*	0.736	0.000*
**Monocytes**	R	0.303	-0.215	-0.341
	*p* value	0.004*	0.103	0.004*
**NE-SSC**	R	-0.397	0.190	0.370
	*p* value	0.000*	0.149	0.002*
**NE-SFL**	R	-0.531	0.021	0.492
	*p* value	0.000*	0.875	0.000*
**NE-FSC**	R	0.096	-0.305	**-**0.233
	*p* value	0.379	0.019*	0.052
**NE-WX**	R	-0.311	0.087	0.289
	*p* value	0.003*	0.511	0.015*
**NE-WY**	R	-0.318	0.083	0.320
	*p* value	0.003*	0.532	0.007*
**NE-WZ**	R	-0.097	0.244	0.247
	*p* value	0.374	0.062	0.039*
**LY-X**	R	-0.573	-0.008	0.482
	*p* value	0.000*	0.950	0.000*
**LY-Y**	R	-0.300	0.097	0.237
	*p* value	0.005*	0.463	0.049*
**LY-Z**	R	-0.458	-0.022	0.411
	*p* value	0.000*	0.871	0.000*
**LY-WX**	R	0.063	-0.070	-0.178
	*p* value	0.562	0.598	0.141
**LY-WY**	R	0.050	0.079	-0.257
	*p* value	0.646	0.550	0.031*
**LY-WZ**	R	-0.263	0.007	0.163
	*p* value	0.014*	0.956	0.181
**MO-X**	R	-0.488	0.099	0.479
	*p* value	0.000*	0.454	0.000*
**MO-Y**	R	-0.258	0.120	0.325
	*p* value	0.016*	0.366	0.006*
**MO-Z**	R	0.236	-0.071	-0.121
	*p* value	0.028*	0.591	0.318
**MO-WX**	R	-0.118	-0.148	0.100
	*p* value	0.278	0.265	0.408
**MO-WY**	R	0.155	-0.024	-0.196
	*p* value	0.152	0.854	0.104
**MO-WZ**	R	-0.051	0.005	0.166
	*p* value	0.643	0.969	0.173

WBCs: white blood cells, NE: neutrophils, LY: lymphocytes, MO: monocytes, * Significant *p* value.

**Fig. 1:**
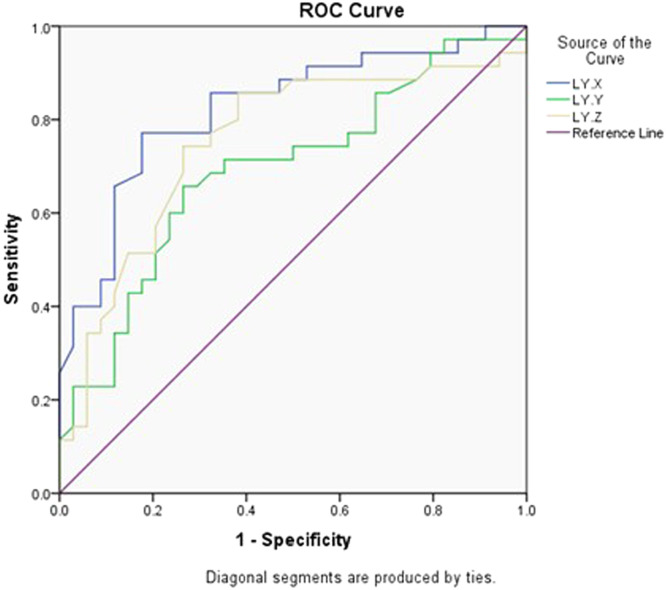
ROC curve analysis of lymphocytes CPD parameters (LY-X, LY-Y, LY-Z) to predict CD4+ *T* cell count <200 cells/mm³.

Using ROC curve analysis, LY-X showed the highest AUC (0.824) compared to LY-Z (AUC= 0.752) and LY-Y (AUC= 0.692) to predict CD4+ *T* cells count <200 cells/mm^3^ (Table 5, Fig. 1).

**Table 5:** ROC curve analysis of prediction of lymphocytes of CD4+ *T* cells <200 cells/mm^3^.

Parameter	AUC	Cut-off	Sensitivity	Specificity
**LY-X**	0.824	78.35	77.1%	82%
**LY-Y**	0.692	68.85	65.7%	73.5%
**LY-Z**	0.752	57.65	74.3%	73.5%

## 4 Discussion

This study analyzed the leucocyte population parameters in HIV and the baseline hematological analysis revealed that PLWH had significantly lower white blood cells, hemoglobin level, and lymphocyte counts than healthy controls, while neutrophil and monocyte absolute counts remained comparable. Previous reports revealed that hematological abnormalities such as cytopenias either anemia, leucopenia, thrombocytopenia, or a combination are frequent in PLWH (Fiseha and Ebrahim, 2022). Lymphopenia underscores the depletion of immune cells characteristic of HIV, in particular the reduction of CD4+ *T* lymphocytes as evidenced by the median CD4+ *T* cell count of 193.95 cells/mm³ in this cohort, indicative of advanced immunosuppression. This is primarily caused by HIV's specific targeting of CD4+ *T*-cells, which express the CD4 receptor and CCR5/CXCR4 co-receptors essential for viral entry (Douek et al., 2003). Although monocytes are less susceptible to HIV infection than lymphocytes, they still become activated upon infection. Their stable numbers in the blood confirm their role as a long-lived viral reservoir that perpetuates immune activation (Veenhuis et al., 2021). Similarly, stable neutrophil counts indicate that HIV-related bone marrow suppression is not severe in this cohort.

Leukocyte CPD provides critical, real-time, objective, and quantitative view of the functional and morphological changes of immune cells. By measuring cell size, granularity, and fluorescence intensity, CPD acts as an early, cost-effective, and rapid diagnostic tool for sepsis, infections, hematological malignancies, and immune responses, offering superior accuracy over manual differentials by tracking the activation state of leucocytes. For example, changes in neutrophil size or granularity as in sepsis reflect the body's immediate immune reaction (Urrechaga, 2020).

Regarding CPD parameters of lymphocytes in this study, increased LY-X in HIV which represents internal cell composition or cell granularity. Similarly, CPD changes were consistent across multiple viral infections, including hepatitis B virus (Zhu et al., 2011), SARS-CoV-2 (Urrechaga et al., 2023), hepatitis C virus, herpes virus, cytomegalovirus, Epstein Barr virus, hepatitis A virus, human herpes virus 8 (Silva et al., 2006), and dengue virus (Hassan et al., 2025). Elevated nucleic acid content and granularity are often hallmarks of cellular activation, immaturity, or stress. The exact molecular mechanisms of HIV to induce changes in leukocytes parameters are still unclear. However, in HIV, chronic immune activation is a cornerstone of disease pathogenesis (Deeks et al., 2004).

Like COVID-19, this persistent state of activation causes morphological changes in immune cells which appear more active or immature on scattergrams. This finding is significant as it quantifies the state of immune activation at a cellular level using routine hematological data. Neutrophils and monocytes are among the first responses to infections. Neutrophil cytoplasmic structure exhibits granulation with increase in nucleic acid contents because of cytokine synthesis. Atypical lymphocytes can be detected in COVID-19. These cells usually present with heterogeneous morphological features including a larger size, a round nucleus often with a large nucleolus, and abundant, deeply basophilic cytoplasm. The volume of monocytes is also altered in activated cells. Similarly, the increase in lymphocyte volume and decrease in lymphocyte conductivity was significantly higher in active infection in relation to immune activation.

In the present study, the observed increased LY-Z, which represents lymphocyte cell size, is explained by the persistent antigenic stimulation by HIV antigens, then lymphocytes enlarge as they enter an activated state, increase their protein synthesis, and prepare for clonal expansion (Coffin and Hughes, 2021). Increased LY-WY which represents dispersion of nucleic acid content, and LY-WZ or the dispersion of cell size in HIV, are among the most significant findings in this study. The increased width or dispersion of these parameters indicates greater heterogeneity within the total lymphocyte population. This likely reflects the presence of multiple lymphocyte subsets in various states of activation, differentiation, and exhaustion (Fenwick et al., 2019; Guo et al., 2023). It may represent a mix of activated HIV-specific CD8+ and CD4+ *T*-cells, in vivo blasts or plasmacytoid cells and anergic or exhausted lymphocytes with different morphological characteristics.

In vitro, the virus-inoculated plasmacytoid dendritic cells developed multinuclear cell syncytia and balloon degeneration (Schmidt et al., 2004). This increased scatter is a direct morphological correlation of the immune dysregulation and high turnover characteristic of chronic HIV infection. In other infections such as HBV, the increase in lymphocyte volume and decrease in lymphocyte conductivity was significantly higher in HBV active infection compared to HBV carrier (Silva et al., 2006).

Regarding the CPD parameters of neutrophils in the current study, the results indicate a highly activated neutrophil state in PLWH. Elevated NE-SSC and NE-SFL suggest increased granularity and a propensity for neutrophil extracellular traps (NETosis), respectively. NETosis is characterized by the release of granule components into cytosol and a modified chromatin (Vorobjeva and Chernyak, 2020). This can be induced by antibodies and immune complexes, cytokines or chemokines such as IL-8 and tumor necrosis alpha (TNF) (Vorobjeva and Pinegin, 2014; Ravindran et al., 2019; Yousefi et al., 2020) which are key contributors to the inflammatory characteristic of HIV pathogenesis. Increased NE-FSC further confirms cellular activation and swelling.

In this study, the relationship with cell size (often indicated by FSC- Forward Scatter) was more complex. CD4+ *T* cells count was negatively correlated with some size-related parameters for lymphocytes (LY-Y, LY-Z) and neutrophils (NE-SFL, NE-WX, NE-WY), suggesting smaller cell size with disease progression, consistent with activation. These changes are consistent with some studies that showed alteration of neutrophils size and internal complexity, consistent with the presence of low density neutrophils (LDNs) and immature forms in the presence of COVID-19 infection reflecting neutrophil degranulation and exhaustion (Manunta et al., 2021).

Monocytes act as reservoirs for HIV and are key drivers of the chronic inflammatory state. The increased complexity (MO-X) suggests changes in their cytoplasmic granularity and organelle content, consistent with an inflammatory state. The broader dispersion (MO-WX) indicates the presence of monocyte subsets (e.g., classical, intermediate, non-classical) with differing morphological characteristics, which is a known feature of HIV where certain inflammatory subsets are expanded. Interestingly, in monocytes, CD4+ *T* cells count was positively correlated with MO-Z (monocytic size). This may indicate a shift in monocyte subsets; a lower CD4+ *T* cells count might be associated with an increase in smaller, perhaps chronically activated or dysfunctional monocytes, or a decrease in larger monocyte subsets, a phenomenon observed in advanced HIV disease (Funderburg et al., 2012).

Despite the presence of opportunistic infections in some cases of this study, the impact of these co-infections on CPD parameters was mainly detected on cellular composition or nucleic acid contents particularly of the neutrophils and monocytes but without impact on cell size. However, these results of the small number of patients with opportunistic infections in the current study should be interpreted cautiously, which requires large-scale studies for confirmation.

In the current study, correlation analysis between standard CBC parameters, CPD, and key HIV biomarkers (CD4+ *T* cells count and viral load) provides an integrated view of the disease's impact on the immune system. The strong and numerous correlations with CD4+ *T* cells count, contrasted with the near-total lack of CD4+ *T* cells count was negatively correlated with parameters like NE-SSC (neutrophil side scatter, indicating granularity/internal complexity), LY-X (lymphocyte nucleic acid content), and MO-X (monocyte nucleic acid content). This suggests that as CD4+ *T* cells counts decline, the remaining lymphocytes, monocytes, and neutrophils exhibit increased internal complexity and nucleic acid content.

Correlation with viral load underscores a critical principle in HIV pathogenesis. The degree of immune deficiency is a more significant determinant of the hematological and cellular state than the absolute level of viral replication at a single time points in untreated infection. However, no significant association was observed with lymphocyte size or morphology, indicating that lymphocyte enlargement and activation are more likely to reflect immune status by CD4+ *T* cells depletion rather than viral burden. Another study described qualitatively that the lymphocyte size (lymphocytes-forward scatter) and reactive lymphocytes (RE-LYMPHO)/leucocytes were higher in COVID-19-positive than negative patients (Martens et al., 2021).

The present study showed that CPD parameters reflecting leucocyte complexity, nucleic acid content, and size were significantly altered in PLWH. Neutrophil and lymphocyte sizes and activation markers were elevated, consistent with immune activation and inflammation inherent in HIV infection. There was also negative correlation of CD4+ *T* cells count with leucocyte morphological parameters, which could be due to immune depletion in progressive disease.

Therefore, the CPD parameters in this study help in stratifying patients' risk beyond just the CD4+ *T* cells count and viral load. This is in concordance with Miyajima et al. who reported the predictive role of CPD in bacteremia in a manner different from that of other indicators (Miyajima et al., 2023).

In our study, ROC curve analysis showed that changes in LY-X which represent lymphocytes complexity predicted advanced HIV better than LY-Y or LY-Z. This is consistent with the significant differences reported of LY-X, LY-Y, neutrophils/lymphocytes ratio, and WBC between moderate and severe groups of COVID-19, and the discrimination power of these CPD markers was confirmed using ROC curve (de Carvalho et al., 2025). These findings collectively suggest that CPD will help in stratifying PLWH who have advanced HIV and guide management such as prophylactic treatment of opportunistic infections.

This study represents the first report on the profile pattern of CDP changes in HIV and its role in clinical stratification. However, limitations exist to the current study. The small sample size and data from single center may limit generalizability of the results. Moreover, lack of comparison with treatment-experienced patients, which could highlight the effect of ART on CPD pattern. In addition, no formal multiple comparison correction was applied to the study and results should be interpreted as exploratory pending confirmation in larger studies. Therefore, further multi-center studies on larger numbers of patients will be recommended.

## 5 Conclusions

HIV causes detectable alterations in leucocyte cell population data. Advanced HIV clinical stages show a significant alteration in complexity and nucleic acid contents of neutrophils and monocytes, and all parameters of lymphocytes. This cost-effective, non-invasive, and practical method can help in assessing progression and immune depletion in PLWH, by providing qualitative and morphometric data on leucocytes as an applicable screening tool for clinical stratification. Further multi-center studies on larger numbers of patients will be recommended to confirm these findings.

## Institutional review board statement

The study was conducted in compliance with the Declaration of Helsinki. The study was also approved by the ethical committee of Faculty of Medicine, Assiut University (IRB no: 04–2023–300268).

## Funding

This work was supported by: Princess Nourah bint Abdulrahman University Researchers Supporting Project (PNURSP2026R148), Princess Nourah bint Abdulrahman University, Riyadh, Saudi Arabia for granting the laboratory work and publishing of this work.

## Data available statement

The data that support the findings of this study are available on a reasonable request from the corresponding author and the data's release adheres to ethical and legal guidelines. The data are not publicly available due to privacy or ethical restrictions.

## Ethics statement

**Institutional Review Board statement:** The study was conducted in compliance with the Declaration of Helsinki. The study was also approved by the ethical committee of Faculty of Medicine, Assiut University (IRB no: 04–2023–300268).

## Informed consent statement

Prior to participation, all enrolled patients provided a written informed consent. Complete confidentiality was maintained when using the participant's medical information.

## CRediT authorship contribution statement

**Haidi Karam-Allah Ramadan:** Formal analysis, Data curation, Conceptualization. **Aml A. Rayan:** Writing – original draft, Methodology, Data curation, Conceptualization. **Zeinab R. Mohamed:** Writing – original draft, Formal analysis, Data curation. **Mohammed Ezz-Eldin:** Methodology, Formal analysis, Data curation. **Ahmed A. Kotb:** Writing – original draft, Formal analysis. **Mohamed Ahmed:** Writing – review & editing. **Rasha Assad Assiri:** Writing – review & editing, Supervision, Funding acquisition. **Salwa Seif Eldin:** Writing – review & editing, Supervision, Funding acquisition. **Amal A. Elkhawaga:** Methodology, Data curation.

## Declaration of competing interest

The authors declare that they have no known competing financial interests or personal relationships that could have appeared to influence the work reported in this paper.

## Appendix Supplementary materials

Image, application 1
