# Supplementary material for: First report on leucocytes cell population data as an applicable test for clinical stratification in people living with HIV
Source: Virus Res. 2026 Mar 20;367:199716. doi: 10.1016/j.virusres.2026.199716 (PMC13053780; doi:10.1016/j.virusres.2026.199716)
Supplement: Supplementary file 1 [file mmc1.docx]

**Supplementary Table 1. Technical description and biological significance of leukocyte cell population data (CPD) parameters measured by the Sysmex XN-1000 analyzer.**

| **Parameter** | **Technical Measurement** | **Biological Significance** |
| --- | --- | --- |
| **NE-SSC** | Neutrophil Side Scatter (X-axis signal) | Reflects internal cell complexity; increases with granules (e.g., toxic granules), vacuoles, and cytoplasmic inclusions. |
| **NE-SFL** | Neutrophil Fluorescence (Y-axis signal) | Reflects cellular nucleic acid (DNA/RNA) content and increases in immature or activated neutrophils (e.g., band cells). |
| **NE-FSC** | Neutrophil Forward Scatter (Z-axis signal) | Reflects the absolute size of the neutrophil population. |
| **NE-WX** | Neutrophils Complexity and the width of dispersion (Width of SSC) | Reflects heterogeneity of internal cellular granularity within the neutrophil population. |
| **NE-WY** | Neutrophils fluorescence and the width of dispersion (Width of SFL) | Reflects heterogeneity of activation within the neutrophil population: dispersion of nucleic acid content. |
| **NE-WZ** | Neutrophils size dispersion Width  (Width of FSC) | Reflects the degree of size heterogeneity of neutrophils population. |
| **LY-X** | Lymphocyte Complexity | Reflects internal complexity; increases with granules or vacuoles (e.g., large granular lymphocytes). |
| **LY-Y** | Lymphocyte Fluorescence | Reflects cellular nucleic acid (DNA/RNA) content and increases in activated or plasmacytoid lymphocytes. |
| **LY-Z** | Lymphocyte Size | Reflects the absolute size of the lymphocyte population. |
| **LY-WX** | Lymphocyte Complexity and the width of dispersion (Width of SSC) | Reflects heterogeneity of internal cellular granularity within the lymphocyte population. |
| **LY-WY** | Lymphocyte fluorescence and the width of dispersion (Width of SFL) | Reflects heterogeneity of activation within lymphocytes population: dispersion of nucleic acid content. |
| **LY-WZ** | Lymphocyte size dispersion Width  (Width of FSC) | Reflects the degree of size heterogeneity of Lymphocytes population. |
| **MO-X** | Monocyte Complexity | Reflects internal cellular complexity and cytoplasmic granularity, which may increase during monocyte activation. |
| **MO-Y** | Monocyte Fluorescence | Reflects cellular nucleic acid (DNA/RNA) content and increases in activated monocytes. |
| **MO-Z** | Monocyte Size | Reflects the absolute size of the monocyte population. |
| **MO-WX** | Monocyte Complexity and the width of dispersion (Width of SSC) | Reflects the degree of granularity heterogeneity: Variation in internal complexity within monocyte population |
| **MO-WY** | Monocytes fluorescence and the width of dispersion (Width of SFL) | Reflects heterogeneity of activation within monocytes population: dispersion of nucleic acid content. |
| **MO-WZ** | Monocyte size dispersion Width  (Width of FSC) | Reflects the degree of size heterogeneity of monocytes population |

NE, neutrophil; LY, lymphocyte; MO, monocyte; SSC, side scatter; FSC, forward scatter; SFL, side fluorescence light; WX, width of side scatter distribution; WY, width of side fluorescence distribution; WZ, width of forward scatter distribution; DNA, deoxyribonucleic acid; RNA, ribonucleic acid.
